# Light-Driven Crystal–Polymer Hybrid Actuators

**DOI:** 10.3389/frobt.2021.684287

**Published:** 2021-05-13

**Authors:** Shodai Hasebe, Daisuke Matsuura, Takaaki Mizukawa, Toru Asahi, Hideko Koshima

**Affiliations:** ^1^ Department of Advanced Science and Engineering, Graduate School of Advanced Science and Engineering, Waseda University, Tokyo, Japan; ^2^ Department of Materials Science and Biotechnology, Graduate School of Science and Engineering, Ehime University, Matsuyama, Japan; ^3^ Research Organization for Nano and Life Innovation, Waseda University, Tokyo, Japan

**Keywords:** actuators, crystal-polymer hybrid, bending motion, light, photoisomerization, salicylideneaniline crystals

## Abstract

Recently, soft robots, which are made of soft and light organic materials, have attracted much attention because of improved safety for daily interactions with humans. Mechanically responsive materials that can move macroscopically by external stimuli, such as light and heat, have been studied extensively over the past two decades, and they are expected to be applicable to soft robots. Among them, mechanically responsive crystals are attractive in terms of a larger Young’s modulus and faster response speed compared with polymers and gels. However, it is impractical to use one piece of a single crystal as a crystal machine; it is difficult to control the size of crystals and obtain large crystals. Hybridization of crystals with polymers is one way to create actuators with more realistic movements. Herein, we report a hybrid crystal assembly in which plate-like salicylideneaniline crystals are aligned in polymer films by a “rubbing” technique, a new approach which is inexpensive, easy, and applicable to a wide range of crystals and polymers. The hybrid films bent reversibly upon alternate irradiation with ultraviolet and visible light. The hybrid films bent as fast as single crystals, even when larger than single-crystal size, showing great mechanical performance originating from the advantages of both molecular crystals (fast response time) and polymers (large size). This work enriches the development of light-driven hybrid actuators composed of molecular crystals and polymers.

## Introduction

Robots are playing increasingly important roles in society. We must consider the symbiotic relationship between humans and robots, as robots may help to improve our lives in the near future. However, conventional robots (hard robots) composed of metals have the disadvantages of being rigid and heavy; they are not suitable for interaction with people from a safety standpoint. More recently, in contrast to hard-bodied robotics, soft robotics has emerged as a new research field (Palagi and Fischer, 2018; Zeng, et al., 2018; Majidi, 2019; Koshima, 2020) because soft-bodied robots (soft robots), which are basically made of organic materials such as polymers and gels, tend to be soft and light and, therefore, more suitable for daily interactions with humans.

Mechanically responsive materials that can move macroscopically by external stimuli, such as light, heat, electricity, chemical reactions, and others, have been extensively studied over the past two decades (Koshima, 2020). Among the various stimuli, light is especially attractive in spatial and temporal selectivity, remote controllability, and variation in its properties (wavelength, intensity, and polarization) (Zeng et al., 2018). Of a significant number of light-responsive materials, liquid crystal polymers (LCPs) containing azobenzene derivatives as mesogens are the most studied materials (White and Broer, 2015; Dong and Zhao, 2018). With different photoresponsive mechanisms such as photochemical liquid crystal (LC)–isotropic phase transition (Yu and Ikeda, 2004), Weigert effect (Yu and Ikeda, 2004), and photothermal effect (Dong and Zhao, 2018), many LCPs that exhibit diverse mechanical motions such as bending (Yu et al., 2003; van Oosten et al., 2009; Cheng et al., 2017), rolling (Yamada et al., 2008; Wie et al., 2016), twisting (Iamsaard et al., 2014), and oscillation (White et al., 2008; Kumar et al., 2016) were reported so far. Besides various photomechanical actuators made of light-responsive LCPs have been developed, such as an inchworm walker (Yamada et al., 2009; Kohlmeyer and Chen, 2013; Lu et al., 2018), a gripper (Lahikainen et al., 2018), a micro swimming robot (Huang et al., 2015; Palagi et al., 2016), a series of photoresponsive fiber arrays for object transport (Gelebart et al., 2016), an iris (Zeng et al., 2017), a microtube that can transfer liquids (Lv et al., 2016), and an artificial flytrap (Wani et al., 2017).

In addition to LCPs, the past decade has witnessed mechanically responsive organic crystals. A crystal is the ultimate solid material, in which molecules arrange regularly in three dimensions. Contrary to their appearance as hard and fragile objects, molecular crystals are attractive as materials that are mechanically responsive to external stimuli. Although the history of research on mechanical molecular crystals is as short as 10 years, many mechanical crystals have been developed that show bending (Kobatake et al., 2007; Koshima et al., 2009; Terao et al., 2012), twisting (Kitagawa et al., 2013; Taniguchi et al., 2016; Kitagawa et al., 2018), rotation (Zhu et al., 2016), jumping (Naumov et al., 2013), locomotion (Taniguchi et al., 2018), and high-frequency bending (Hagiwara et al., 2020). In terms of mechanical properties, crystals have the advantage of a larger Young’s modulus (Taniguchi et al., 2019) than polymers and gels as well as a faster response to external stimuli (Naumov et al., 2020); this has motivated the development of crystal soft robots composed of mechanically responsive crystals. It is, however, impractical to use a single crystal piece as a crystal machine, yet it is difficult to control the size of crystals and obtain crystals with sizes ranging from a few centimeters to several tens of centimeters.

Hybridization of crystals with polymers is one approach to create actuators with more realistic movements. Such hybrid materials are promising as actuators due to fabrication simplicity and ease of size and shape control. Hybrid photomechanical membranes, in which nanorod crystals are assembled with poly (vinylidene fluoride–co-hexafluoropropylene) polymers, have been reported (Lan and Chen, 2013). We have reported a hybrid crystal assembly in which plate-like salicylideneamine crystals are aligned in silicone polymer films by a magnetic field; the polymer films bent toward the light source upon exposure to ultraviolet (UV) light and returned to the original straight shape reversibly upon exposure to visible light (Koshima et al., 2013). However, the alignment of crystals by a magnetic field has the drawback that only a limited number of crystals could be oriented by a magnetic field; for the purpose of expanding the versatility of crystal–polymer hybrid materials, new alignment methods are necessary.

**FIGURE 1:**
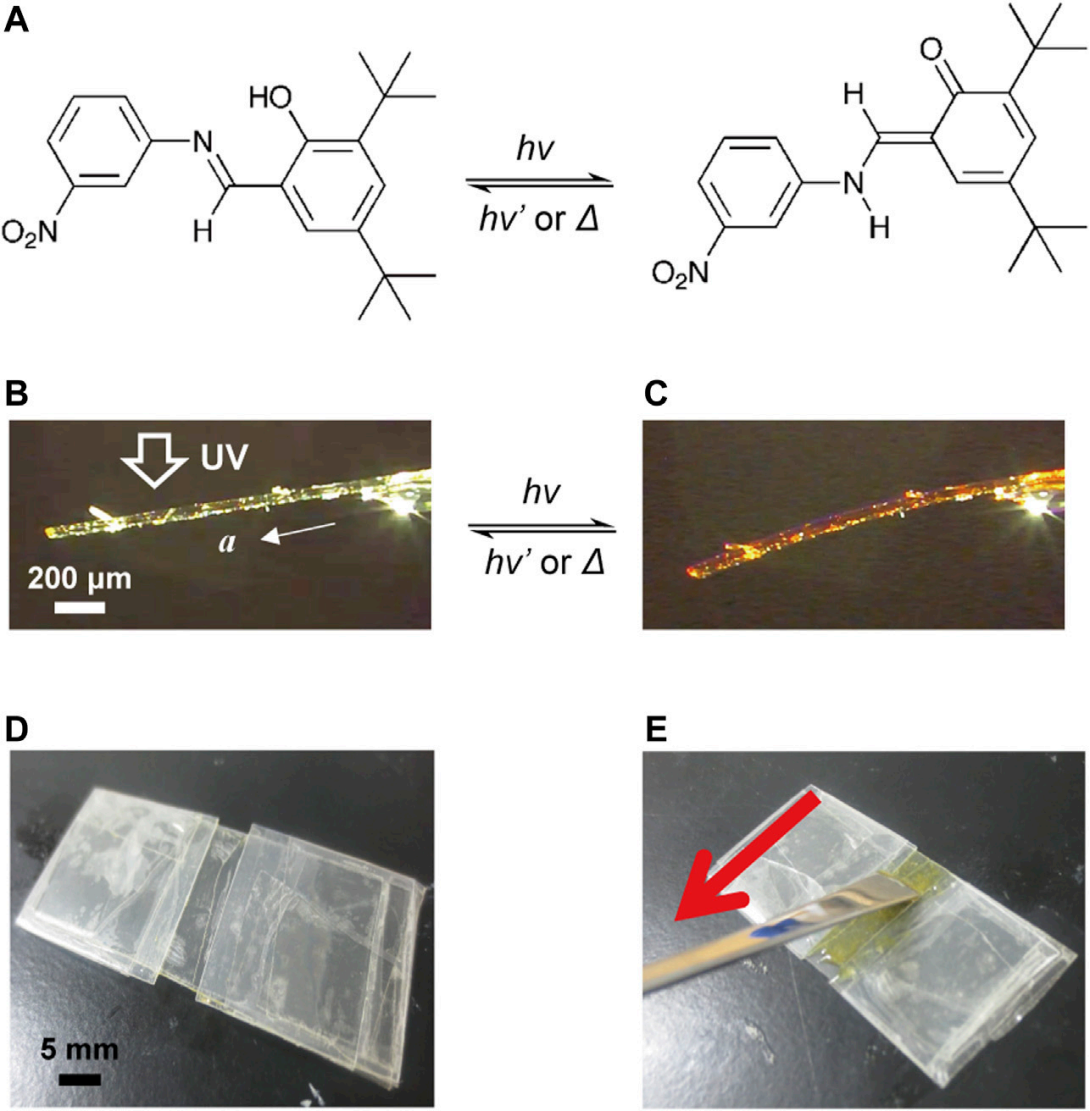
**(A)** Enol–keto photoisomerization of salicylideneaniline 1. **(B,C)** Bending of a plate-like enol-**1** crystal (1410 μm long × 95 μm wide × 47 μm thick) **(B)** before and **(C)** after UV light irradiation from the top side. **(D)** A shallow groove mold made of polypropylene films. **(E)** A “rubbing” technique in which the surface of polymer suspension in the mold is stroked with a spatula to align the crystals with the long direction along the rubbing direction.

In a previous report, we demonstrated that the plate-like crystals of *N*-3,5-di-*tert-*butylsalicylideneaniline-3-nitroaniline (1) in enol form reversibly bent away from the light source under UV light exposure and returned to the original straight shape when illuminated with visible light (Koshima et al., 2011) (Figures 1A–C). In this work, we report a hybrid crystal assembly in which plate-like enol-**1** crystals are aligned in a silicone polymer film and UV-cured resin film by a “rubbing” technique. The hybrid films bent reversibly upon alternate irradiation with UV and visible light, and we revealed that the hybrid films bent as fast as single crystals, even at sizes larger than a single crystal (Koshima et al., 2011).

## Materials and Methods

### Sample Preparation

The compound enol-**1** was synthesized according to the literature (Smith et al., 1964). Plate-like enol-**1** crystals having length ranging from 50 to 500 μm were obtained by recrystallization from 2-propanol solution. A shallow groove mold (20 mm long, 5 mm wide, 150 μm thick) was prepared by placing two pieces of polypropylene film in parallel on a polypropylene sheet (Figure 1D).

The silicone polymer hybrid film was prepared as follows. The suspension (20%) of the enol-**1** crystals (0.1 g) in the liquid silicone precursor (0.4 g; TSE387; Momentive Performance Materials, Japan) was poured into the mold, and the surface was gently rubbed several times with a spatula in the direction of the groove length to align the long axis of the crystal to the stroking direction (Figure 1E). Then, the suspension was heated overnight at 60°C to solidify the precursor, giving a rubbery elastic film.

The UV-cured resin hybrid film was prepared as follows. The suspension (20%) of the enol-**1** crystals (0.1 g) in the acrylate-based UV-curable monomer liquid (0.4 g; UV Curing Resin “Sun Drop” [Hard Type]; PADICO Co., ltd., Japan) was poured into the mold. In the same way as silicon polymer hybrid films, the surface of the suspension in the mold was gently rubbed several times with a spatula in the direction of the groove length to align the long axis of the crystal to the rubbing direction (Figure 1E). Then, the surface was irradiated with UV light (365 nm, 180 mW cm^−2^) for 1 min to give a UV-cured hybrid film.

**FIGURE 2:**
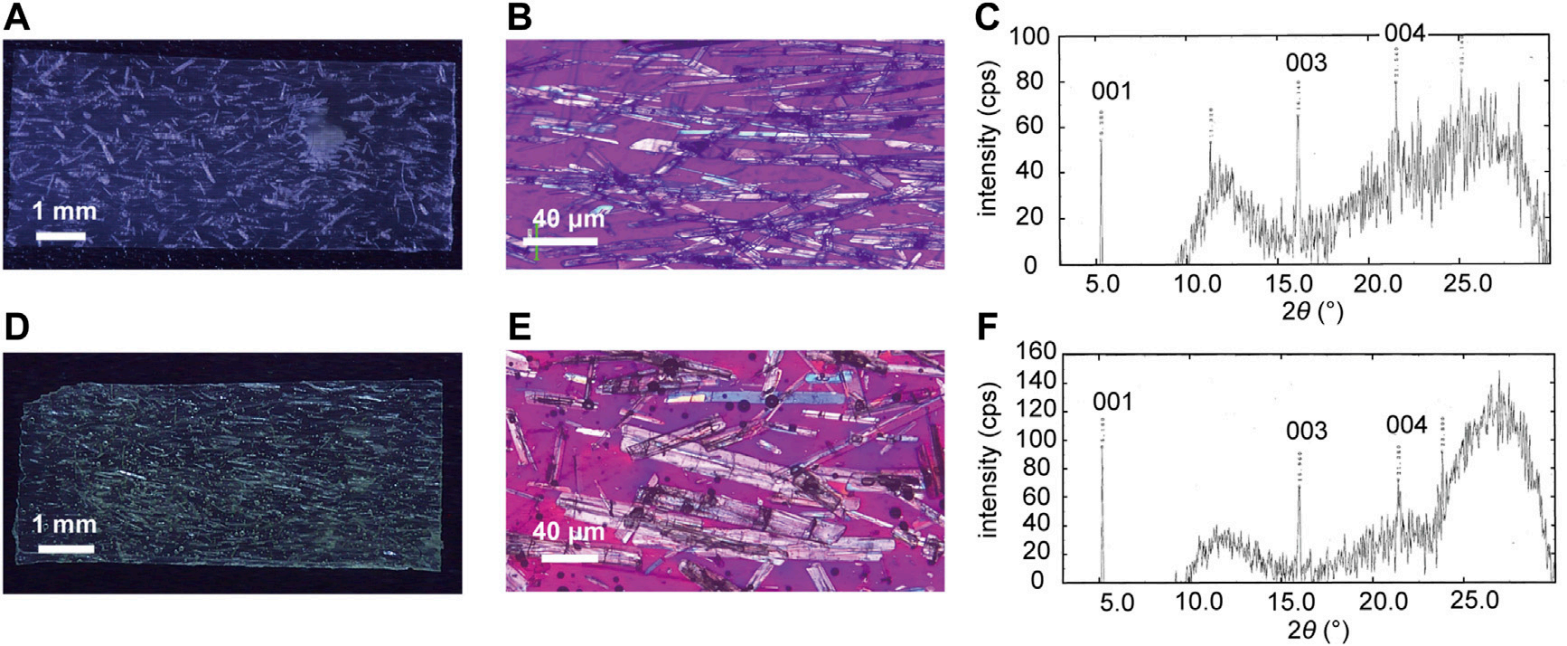
**(A,D) (A)** A silicone polymer hybrid film (8.6 mm long × 3.7 mm wide × 100 μm thick) and **(D)** a UV-cured resin hybrid film (7.0 mm long × 3.0 mm wide × 126 μm thick) in which enol-**1** crystals are aligned by rubbing. **(B,E)** Enlarged polarized microscopic images of **(B)** the silicone polymer hybrid film and **(E)** the UV-cured resin hybrid film. **(C,F)** XRD profiles of **(C)** the silicone polymer hybrid film and **(F)** the UV-cured resin hybrid film.

The plate-like enol-**1** crystals were almost completely aligned along the long axis, parallel to the stroking direction of the suspension surface by the spatula. The top surface of the crystals was identified as the (001) face by X-ray diffraction (Figures 2C,F), with the long direction along the *a* axis based on comparisons with plate-like bulk crystals (Koshima et al., 2011).

### Absorption Spectra

Ultraviolet–visible (UV–vis) absorption spectra were measured with a conventional optical microscope equipped with an optical fiber connected to a CCD spectrometer (Exemplar Plus LS), measurement light (a xenon lamp) and a temperature-controller stage (LMT-600S; Collet Kogyo Co., Ltd.). Transmitted light intensity through the sample was measured between 360 and 800 nm at 1 nm resolution with an acquisition time of 100 ms. To reveal photoisomerization properties, the sample was irradiated with a UV laser (375 nm; FOLS-03; Sawaki Kobo) and after the spectral change was saturated, the UV source was turned off and the sample was exposed to a visible light laser (520 nm; FOLS-02; Sawaki Kobo).

### Observation of Mechanical Motion

The hybrid film subjected to bending was fixed between two pieces of glass. Ultraviolet irradiation (365 nm, 180 mW cm^−2^, spot diameter 8 mm) was applied to the hybrid films using a ultraviolet–light-emitting diode (UV-LED) irradiator (Keyence UV-400). Visible light (530 nm) was illuminated using a xenon-lamp irradiation system (Asahi Spectra MAX-302) through a bandpass filter. Photomechanical motion of hybrid films upon irradiation was recorded by a digital high-speed microscope (Keyence VW-6000) fitted with a zoom lens. Movies were analyzed using Tracker Video Analysis and Modeling Tool software (Brown, 2019).

## Results and Discussion

### Characterization of Hybrid Films

Figures 2A,B show the silicone polymer hybrid film in which the plate-like enol-**1** crystals of length ranging from 50 to 500 μm are almost completely aligned along the long axis, parallel to the rubbing direction of the suspension in the shallow groove mold. X-ray diffraction measurements of the film revealed three peaks (Figure 2C), which were assigned to the 001, 003, and 004 reflections based on comparison with published crystallographic data (Koshima et al., 2011). The top surface of the plate-like enol-**1** crystals was identified as the (001) face, with the long direction along the *a* axis based on comparisons with other plate-like bulk crystals. The UV-cured resin hybrid film also gave the crystal orientation almost completely aligned in the length direction, and the crystal top surface corresponded to the (001) plane (Figures 2D–F).

We then measured the UV–visible absorption spectra of a UV-cured resin film to reveal the enol–keto photoisomerization of the enol-**1** crystals hybridized in the UV-cured film. Supplementary Figure S1B presents the spectral changes of the UV-cured hybrid film upon UV (375 nm, 90 mW cm^−2^) irradiation. Before UV illumination, the film absorbed weakly at wavelengths longer than 550 nm and exhibited a tail to wavelengths longer than 450 nm. Upon UV irradiation, the absorption at 400–600 nm increased and reached steady-state after 60 s. The difference spectrum, obtained by subtracting the spectra obtained before and after UV irradiation, exhibited an absorption peak at 485 nm due to photoisomerization from the enol to the *trans*-keto form (Supplementary Figure S1D). The time constant of the photoisomerization, an indicator of the rate of the reaction (the time for the reaction to reach (1–1/e) ≈ 63.2% of its final state’s value), was about 7.1 s. Immediately after turning off the UV light, the hybrid film was illuminated by visible light (520 nm, 62 mW cm^−2^) (Supplementary Figure S1F); the absorption peak at 485 nm decreased due to photochemical back-isomerization of the *trans*-keto to the enol form, and reached the original state after 3 s. The time constant of photochemical back-isomerization was 1.4 s. A single enol-**1** crystal also exhibited similar photoisomerization properties upon UV light irradiation and subsequent visible light exposure (Supplementary Figures S1A,C,E) with comparable time constants in bending (16.9 s) and straightening (1.2 s). These results confirm that the enol-**1** crystals hybridized in the UV-cured resin film, like single enol-**1** crystals, underwent enol–keto photoisomerization upon UV light exposure and keto–enol photochemical back-isomerization under subsequent visible light irradiation.

### Light-Driven Bending of Hybrid Films

**FIGURE 3:**
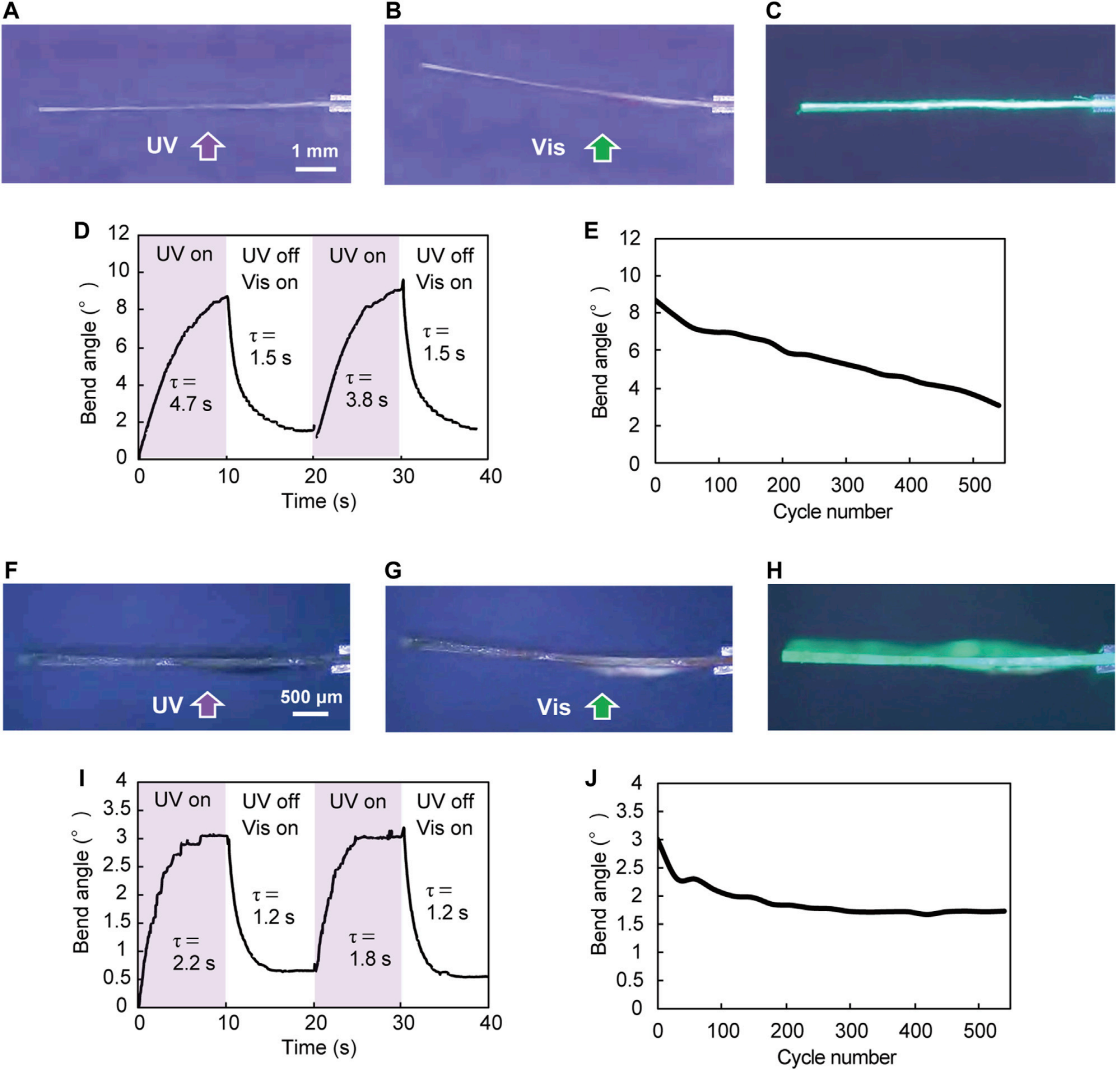
Photomechanical bending of hybrid films upon alternate irradiation with UV-LED (365 nm, 180 mW cm^−2^, spot diameter 8 mm) for 10 s and visible light (530 nm, 10 mW cm^−2^) for 10 s. **(A–E)** Photomechanical bending of a silicone polymer hybrid film (8.6 mm long × 3.7 mm wide × 100 μm thick) (Supplementary Movie S1). **(A**–**C)** UV irradiation of the film from the lower side, and side view of photomechanical bending of the film **(A)** before, **(B)** after UV irradiation for 10 s, and **(C)** subsequent illumination with visible light for 10 s. **(D)** Time dependence of bend angle (two cycles) and **(E)** repeatability of the reversible bending over 550 cycles. **(F**–**J)** Photomechanical bending of a UV-cured resin hybrid film (7.0 mm long × 3.0 mm wide × 126 μm thick) (Supplementary Movie S2). **(F**–**H)** UV irradiation of the film from the lower side, and side view of photomechanical bending of the film **(F)** before, **(G)** after UV irradiation for 10 s, and **(H)** subsequent illumination with visible light for 10 s. **(I)** Time dependence of bend angle (two cycles) and **(J)** repeatability of the reversible bending over 550 cycles.

The silicone polymer hybrid film in which plate-like enol-**1** crystals were aligned by rubbing was cut into a rectangle (8.6 mm long × 3.7 mm wide × 100 μm thick; Figure 2A, Figure 3A). One end of the film was fixed between two glass plates. When the film was irradiated from the lower side with the UV-LED light (365 nm, 180 mW cm^−2^, spot diameter 8 mm; Figure 3A), the film bent away from the light to reach a bend angle of 8.7° after 10 s (Figure 3B, Supplementary Movie S1). Subsequent UV stopping and, instead, visible light illumination with a xenon lamp through a color filter (530 nm, 10 mW cm^−2^) returned the bend angle to almost its initial flat shape (1.5°) after 10 s (Figure 3C); the bend angle time profile is shown in Figure 3D; the time required for bending and straightening was comparable to that of molecular crystals (5–30 s for bending and 5–40 s for straightening, Koshima et al., 2011), suggesting that the influence of the stiffness of the silicone polymer on the bending under light irradiation is relatively small. The time constants for bending and straightening were 4.7 s and 1.5 s, respectively. It took, in contrast, 1,350 s for the recovery of flat form after turning off the UV light without subsequent visible light illumination; this was because thermal back-isomerization proceeded more slowly than photochemical back-isomerization (Koshima et al., 2011). The bend motion of the silicone polymer hybrid film was repeatable; upon alternate irradiation of UV (10 s) and visible light (10 s), the film repeated its bending and straightening motions. The bend angle gradually decreased from 8.7° to 3.1° over 550 cycles, demonstrating good durability (Figure 3E).

The UV-cured resin hybrid film in which plate-like enol-**1** crystals were aligned by rubbing was also cut into a rectangle (7.0 mm long × 3.0 mm wide × 126 μm thick; Figures 2D, 3F). When the film was irradiated from the lower side with a UV-LED light (365 nm, 180 mW cm^−2^, spot diameter 8 mm), the film bent away from the light to reach a maximum bend angle of 3.0° after 7 s, and then maintained a steady angle (Figure 3G, Supplementary Movie S2). Subsequent stopping of the UV light and illuminating with visible light (530 nm, 10 mW cm^−2^) returned the bend to almost its initial flat shape (0.6°) after 5 s, and then maintained a steady angle (Figure 3H). The bend angle time profile is shown in Figure 3I; as with the silicone polymer film, the time required for bending and straightening was comparable to that of molecular crystals, showing that the influence of the stiffness of the UV-cured resin on the bending under light irradiation is relatively small. The time constants for bending and straightening were 2.2 s and 1.2 s, respectively, nearly coincident with the time constants of 7.1 s and 1.4 s in photoisomerization and photochemical back-isomerization (Supplementary Figures S1D,F). It took, on the other hand, 106 s for recovery of the flat form after UV stopping without subsequent visible light illumination because of slow thermal back-isomerization. The UV-cured resin film also exhibited repeated bending. Upon alternate irradiation of UV (10 s) and visible light (10 s), the film showed continuous bending and straightening motions; the bend angle gradually decreased from 3° to 1.7° up to 300 cycles, and then remained constant over 550 cycles, demonstrating excellent durability (Figure 3J).

The bending motion of the UV-cured resin hybrid films depended on the intensity of UV light. When a hybrid film (25 mm long × 3.8 mm wide × 136 μm thick) was irradiated at 365 nm at 45–180 mW cm^−2^ for 10 s, the bend angle increased from 0° to 1.8°, respectively, in proportion to the UV intensity (Supplementary Figure S2). We also revealed that the bend angle decreased in proportion to thickness of the film. The maximum bend angles of 3.0°, 1.8°, 0.40°, and 0.15° were observed with hybrid films having thicknesses of 126 μm, 136 μm, 153 μm, and 238 μm, respectively, by UV light irradiation (365 nm, 180 mW cm^−2^) for 10 s (Supplementary Figure S3).

**FIGURE 4:**
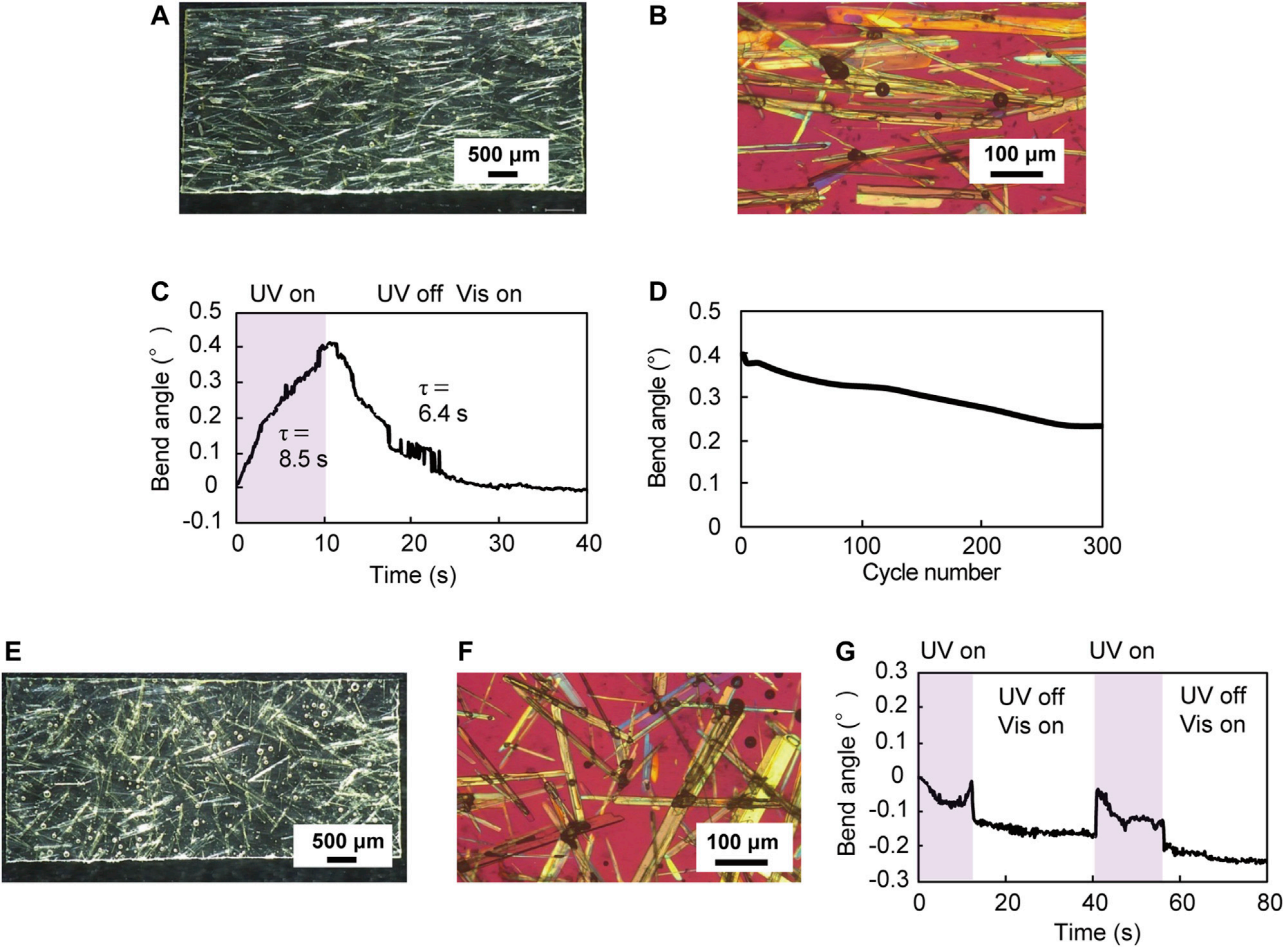
**(A)** A UV-cured resin hybrid film (7.1 mm long × 3.2 mm wide × 153 μm thick) in which enol-**1** crystals are aligned by rubbing. **(E)** A UV-cured resin hybrid film (7.0 mm long × 3.2 mm wide × 155 μm thick) in which enol-**1** crystals are not aligned. **(B,F)** Enlarged polarized microscopic images of **(B)** the film in which enol-**1** crystals are aligned and **(F)** the film in which enol-**1** crystals are not aligned. **(C,G)** Time dependence of bend angle upon UV-LED irradiation of **(C)** the film in which enol-**1** crystals are aligned and **(G)** the film in which enol-**1** crystals are not aligned. (**D**) Repeatability of the reversible bending of the aligned film over 300 cycles.

Furthermore, we investigated the effect of crystal alignment on the mechanical behavior of the hybrid film by comparing the mechanical behavior of a UV-cured resin hybrid film in which plate-like enol-**1** crystals were aligned with a film in which the crystals were not aligned. Figures 4A,B show a UV-cured resin hybrid film (7.1 mm long × 3.2 mm wide × 153 μm thick) in which enol-**1** crystals were aligned by the “rubbing” technique. The UV-cured films in Figure 4 were thicker (∼150 μm) than that of the UV-cured film shown in Figure 3 (126 μm), probably due to the difference in rubbing strength. Upon UV-LED irradiation (365 nm, 180 mW cm^−2^, spot diameter 8 mm), the film bent away from the light source with a maximum bend angle of 0.4° in 10 s (Figure 4C). After ceasing UV light irradiation, and under visible light (530 nm, 10 mW cm^−2^) illumination, the film returned to the original straight shape in 20 s. The time constants for bending and straightening were 8.5 s and 6.4 s, respectively. The maximum bend UV-cured resin film shown in Figures 3F–J (7.0 mm long × 3.0 mm wide × 126 μm thick), probably due to the greater film thickness (Supplementary Figure S3). The bend motion was repeatable; upon alternate irradiation of UV (10 s) and visible light (30 s), the film showed continuous bending and straightening motions over 300 cycles (Figure 4D). Figures 4E,F show a UV-cured resin hybrid film (7.0 mm long × 3.2 mm wide × 155 μm thick) in which enol-**1** crystals were not aligned. Unlike the aligned film shown in Figures 4A–D, this film did not bend away from the light source upon UV light irradiation (Figure 4G); instead, the film bent slightly toward the light source. Considering that enol-**1** crystals bend away from the light source by photoisomerization (Figures 1A–C), these results indicate that aligning of the crystal orientation is essential to bend films away from the light source via photoisomerization of crystals.

## Conclusion

Silicone polymer film and UV-cured resin film in which plate-like salicylideneaniline crystals were aligned were prepared by a “rubbing” technique, a new approach which is inexpensive, easy, and applicable to a wide range of crystals and polymers. Upon UV light irradiation, the hybrid films reversibly bent away from the light source and returned to the original straight shape upon subsequent irradiation with visible light. The speed in bending was comparable to that of single crystals, even at larger than single-crystal size, demonstrating great mechanical performance originating from the advantages of both molecular crystals (fast response time) and polymers (large size). The results provide insight into light-driven hybrid actuators composed of molecular crystals and polymers and suggest new approaches for the development of soft robots. The mechanical properties such as Young’s modulus, mechanical forces, and the relationship between the film size, shape, and the crystal amount and the bending behavior should be discussed in future. Precise control of the thickness of the hybrid films is also an issue for the future.

## Data Availability

The original contributions presented in the study are included in the article/Supplementary Material, further inquiries can be directed to the corresponding author.
