# Supplementary material for: Light-Driven Crystal–Polymer Hybrid Actuators
Source: Front Robot AI. 2021 May 13;8:684287. doi: 10.3389/frobt.2021.684287 (PMC8155379; doi:10.3389/frobt.2021.684287)
Supplement: Supplementary file 1 [file DataSheet1.pdf]

Supplementary Material

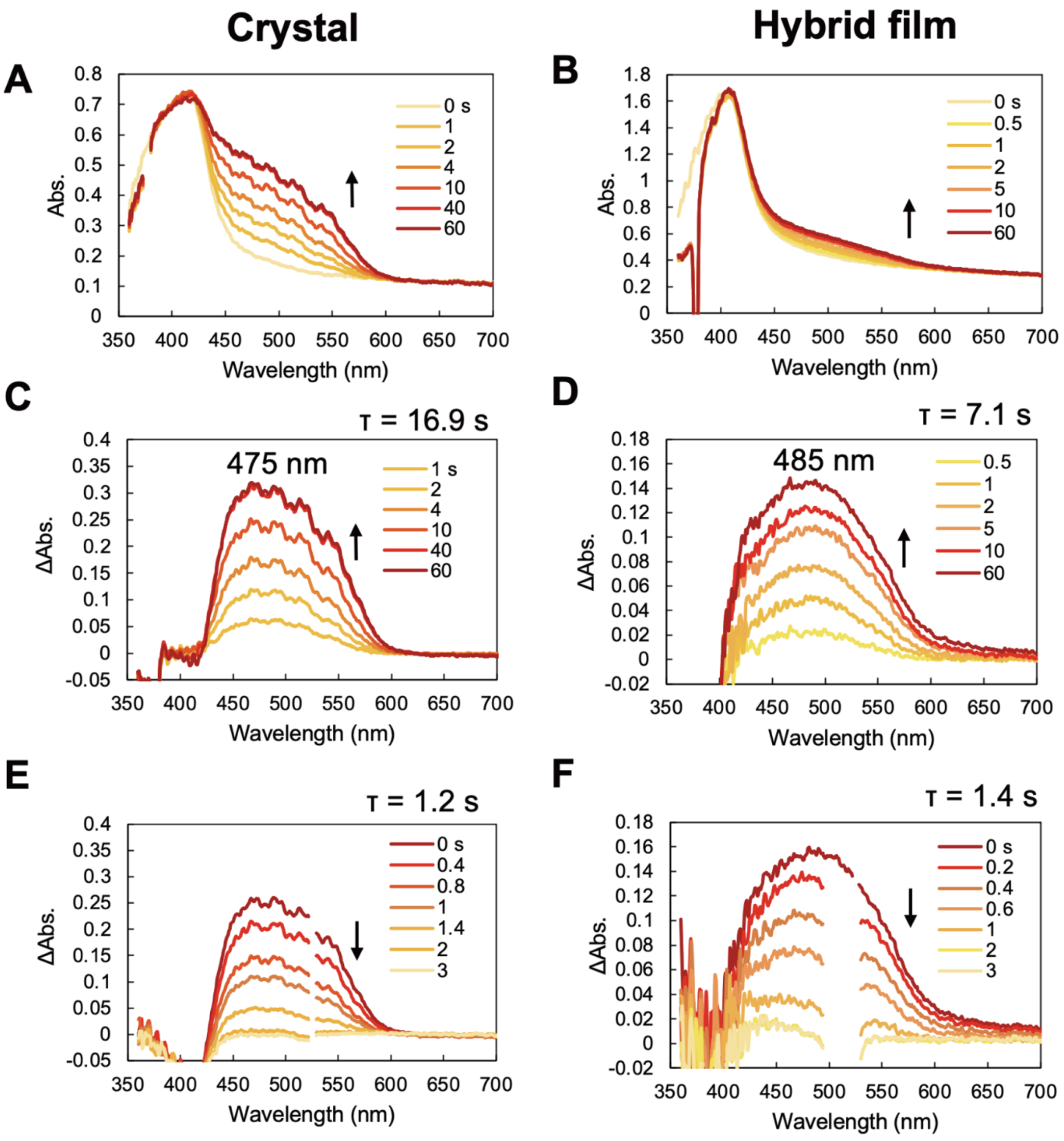

Figure S1 | (A, B) Absorption spectra and **(C–F)** absorption difference spectra of **(A, C, E)** a single enol-**1** crystal, and **(B, D, F)** a UV-cured resin hybrid film in which enol-**1** crystals are aligned. **(A–D)** UV irradiation (375 nm, 90 mW cm^{-2}), and then **(E, F)** visible light irradiation (520 nm, 62 mW cm^{-2}).

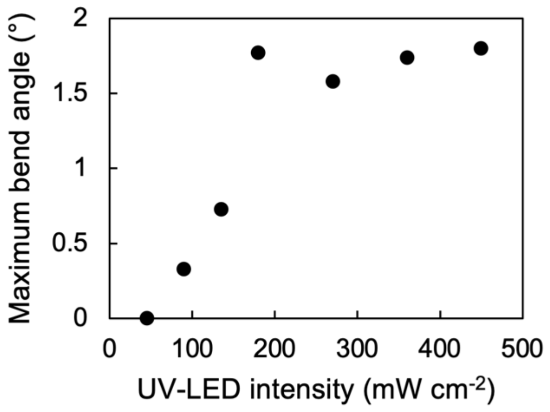

Figure S2 | Light intensity dependence of maximum bend angle of a UV-cured resin hybrid film (25-mm long × 3.8-mm wide × 136-μm thick) upon UV-LED (365 nm) irradiation for 10 s.

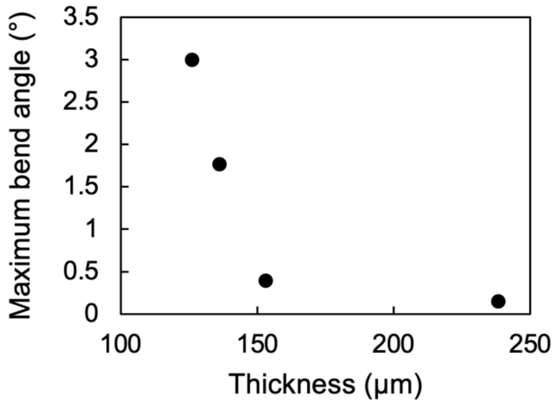

Figure S3 | Thickness dependence of maximum bend angle of UV-cured resin hybrid films (7.0-mm long × 3.0-mm wide × 126-μm thick), (25-mm long × 3.8-mm wide × 136-μm thick), (7.1-mm long × 3.2-mm wide × 153-μm thick), and (18-mm long× 3.0-mm wide × 238-μm thick) upon UV-LED (365 nm, 180 mW cm^{-2}) irradiation for 10 s.

List of Movies

Movie S1 | Photomechanical bending of a silicone polymer hybrid film (8.6 mm long × 3.7 mm wide × 100 μm thick) upon UV-LED (365 nm, 180 mW cm^{-2}, spot diameter 8 mm) irradiation for 10 s and subsequent illumination with visible light (530 nm, 10 mW cm^{-2}) for 10 s (Realtime).

Movie S2 | Photomechanical bending of a UV-cured resin hybrid film (7.0 mm long × 3.0 mm wide × 126 μm thick) upon UV-LED (365 nm, 180 mW cm^{-2}, spot diameter 8 mm) irradiation for 10 s and subsequent illumination with visible light (530 nm, 10 mW cm^{-2}) for 10 s (Realtime).
